# Genotype analysis to clarify RhD variants in discrepant samples of Chilean population

**DOI:** 10.3389/fimmu.2023.1299639

**Published:** 2023-12-05

**Authors:** Andrés Aburto, Diego Zapata, Eduardo Retamales, Jorge Fernández, Gisselle Barra, Francisca Peña, Sofía Cárcamo, Nicolás Saavedra, Cristian Sandoval, Juan Orellana, José Caamaño

**Affiliations:** ^1^ Sección Hematología e Inmunohematología, Departamento Laboratorio Biomédico Nacional y de Referencia, Instituto de Salud Pública de Chile, Santiago, Chile; ^2^ Subdepartamento de Genética Molecular, Departamento Laboratorio Biomédico Nacional y de Referencia, Instituto de Salud Pública de Chile, Santiago, Chile; ^3^ Laboratorio de Inmunohematología y Medicina Transfusional, Departamento de Medicina Interna, Facultad de Medicina, Universidad de La Frontera, Temuco, Chile; ^4^ Centro de Investigación en Medicina de Laboratorio – CeMLab, Facultad de Medicina, Universidad de La Frontera, Temuco, Chile; ^5^ Departamento de Ciencias Básicas, Facultad de Medicina, Universidad de La Frontera, Temuco, Chile; ^6^ Escuela de Tecnología Médica, Facultad de Salud, Universidad Santo Tomás, Osorno, Chile; ^7^ Departamento de Ciencias Preclínicas, Facultad de Medicina, Universidad de La Frontera, Temuco, Chile; ^8^ Departamento de Salud Pública, CIGES (Capacitación, Investigación y Gestión para la Salud), Facultad de Medicina, Universidad de La Frontera, Temuco, Chile

**Keywords:** blood, blood transfusion, Coombs test, population groups, Rh-Hr blood-group system

## Abstract

**Introduction:**

The D antigen variants are classified as weak, partial, and extremely weak (DEL) and can be differentiated using molecular tests. In Chile, the laboratories of local blood centers do not identify variants of the D antigen, referring them for study to the Reference Laboratory of the Public Health Institute of Chile. So, our aim was to talk about the results of the molecular analysis of variants of the D antigen in samples that had different results in the serological classification.

**Methods:**

In the D antigen classification of the Rh system, 479 samples with serological discrepant results were sent for molecular analysis. The Rh phenotype was performed with monoclonal anti-C, anti-c, anti-E, and anti-e antisera by direct agglutination. To find the D antigen, researchers used direct agglutination with monoclonal antisera and indirect antiglobulin testing with the column (gel) agglutination method. Molecular analysis was performed with a polymerase chain reaction with sequence-specific primers (SSP-PCR) and sequencing.

**Results and discussion:**

The presence of D antigen variants was confirmed in 332 samples (69.3%), with an initial discrepancy in serological classification. In this group of discrepant samples, the frequency of weak RhD variants was 66% (219/332), that of extremely weak RhD was 28% (93/332), and that of partial RhD was 6% (20/332). The weak variants type 2 (27.4%), type 3 (8.4%), type 48 (8.4%), and type 1 (8.1%) were the next most prevalent variants after RHD*DEL43 (28%). The ccEe (R2r) phenotype was the most frequently detected (38.4%) and is present in 87% of the RHD*DEL43 samples. The E antigen is associated with the presence of this variant. Our analyses give the first description of D antigen variants in Chile. The most common variants are DEL type (RHD*DEL43) and weak (weak type 2), which are linked to the ccDEe (R2r) phenotype. These findings allow us to characterize the variants of the D antigen in Chile and, according to the obtained data, to design strategies for the management of donors, patients, and pregnant women.

## 1 Introduction

After the ABO system, the Rh blood group has great importance in transfusion medicine since it corresponds to one of the most complex, polymorphic, and immunogenic among the human blood groups. These antigens are made by the RHD and RHCE genes, which are homologous and are on the short arm of chromosome 1 (p34–36). They are arranged in opposite directions and are separated by the TMEM50A gene (1). There are 10 exons in each of these genes that code for RhD and RhCE proteins, which are different by 32 to 35 amino acids out of a total of 416 amino acids (2).

On a routine basis, blood might be serologically classified as Rh positive (D+) or Rh negative (D-), depending on the presence or absence of D antigen on the red blood cells (RBCs) membrane. The complete deletion of the RHD gene is the most common cause of the RhD-negative phenotype (3, 4). However, D antigen can exhibit qualitative and quantitative alterations as a result of variations affecting its DNA sequence, i.e., due to a 37-bp internal duplication (5), in which RhD is more difficult to detect and constitutes a relevant issue in transfusion medicine since the exposure of RhD-treated subjects to RhD+ cells could produce anti-D alloantibody, which in turn might cause hemolytic disease of the fetus and newborn (HDFN) and transfusion reactions (6). These RhD variants might be grouped into partial, weak, and extremely weak variants (DEL). Partial D are a type of qualitative variants. It happens when the D antigen is missing one or more epitopes because of a change in the DNA sequence that codes for the antigen’s extracellular domains. Some monoclonal antibodies may be able to detect the presence of this variant, and carrying individuals may develop anti-D antibodies following exposure to wild-type D through transfusion or pregnancy (1). Weak variants are changes in the amount of RhD antigen in the red blood cells. This happens when mutations cause amino acid changes in the transmembrane or cytoplasmic domains, but the conformation of the epitope stays the same (7). By using the indirect antiglobulin test (IAT), routine serology can detect partial and weak variants. However, the DEL variant has a very weak expression of the D antigen that the IAT cannot detect. Because of this, people with the DEL variant are called RhD-. This variant can be detected serologically by non-routine techniques using adsorption and elution studies (1, 8).

The frequency of the RhD negative phenotype varies among different ethnic groups, with a frequency of approximately 15% in Caucasians and <0.5% in Eastern Asians (9, 10). There is a negative frequency of 5.5% for RhD in the Chilean population, but no one knows how common D variants are or what DNA changes are linked to these D variants (11). Thus, the aim of the present study was to describe the RhD variants present in a group of serologically discrepant samples obtained from the Reference Laboratory of the Public Health Institute of Chile.

## 2 Materials and methods

### 2.1 Samples

A total of 479 EDTA samples from patients and blood donors from Chilean blood services (blood centers, transfusion medicine units, and laboratories), both public and private, were evaluated. Samples met some of the following criteria: i) RhD discrepancy in serological classification, assessed through results with DVI+ and DVI-antisera; ii) low reactivity in DVI+ and DVI- antisera (ideally ≤2+); iii) the RhD phenotype cannot be confirmed because the sample is Direct Coombs positive; iv) and RhD discrepancy between different blood services (RhD+ reported by one laboratory and RhD- reported by another laboratory for the same patient). The samples were derived between 2014 and 2020 for the study of weak and partial D variants at the Immunohematology Reference Laboratory of the Public Health Institute of Chile. This study retrospectively reviews the medical records of patients who have undergone a specific procedure for clinical purposes.

### 2.2 RhD serological phenotyping

The D antigen was found with anti-D IgM clones ESD-1M/175-2 and LHM59/20 (LDM3)/175-2 on a gel card (ID-Card DiaClon ABO/D, Bio-Rad Laboratories, CA, USA). Then, the resulting D- samples were analyzed by IAT (ID-Card LISS/Coombs, Bio-Rad Laboratories, CA, USA) using manual and automated methods (IH-500 System, Bio-Rad Laboratories, CA, USA) with anti-D IgG clone ESD1 and anti-D IgG/IgM clones MS26/TH28.

### 2.3 Rh CE phenotyping

We studied the phenotypes of RhC/c and RhE/e antigens by directly agglutinating them in saline using the gel card system (Diaclon Rh-Subgroups + C^w^ + K, Bio-Rad Laboratories, CA, USA) and monoclonal antibodies against C, c, E, and e by manual and automated methods (IH-500 System, Bio-Rad Laboratories, CA, USA). The antibodies used were anti-C IgM clone MS-273/P3x25513G8; anti-c IgM clone 951; anti-E IgM clone MS-80/MS-258; and anti-e IgM clone MS-62/P3GD512.

### 2.4 Molecular RHD analysis

Genomic DNA was extracted from blood samples using a commercially available DNA extraction kit (Dneasy Blood & Tissue Kit; Qiagen Inc., CA, USA), following the manufacturer’s protocol. The RHD and RHCE gene polymorphisms were found using sequence-specific primer polymerase chain reaction (SSP-PCR) with RH-type, partial D-type, and weak D-type kits (BAG Diagnostics, GmbH, Lich, Germany). This method detects all ten exons of *RHD* and types C, c, E, and e of the *RHCE* gene according to the manufacturer’s procedures. Results were expressed as positive or negative according to the presence or absence of specific PCR products in each kit, whose interpretation pattern will define each genotype. An internal amplification control is included in each reaction mix.

For the samples that were inconclusive by the PCR method, RHD gene sequencing was performed. Legler et al.’s primer descriptions served as the basis for PCR amplification (12). The amplicons generated were sequenced in both directions by using a BigDye Terminator Cycle Sequence Kit v3.1 (Applied Biosystems, Foster City, CA, USA). Nucleotide sequences were obtained using an ABI PRISM 3500 Genetic Analyzer (Applied Biosystems). The sequence data generated were assembled and edited electronically using the Sequencher and EDITSEQ programs.

We used the reference sequence NG_007494.1 from the NCBI website and compared it to the sequences from the samples to find the mutations that were there (https://www.ncbi.nlm.nih.gov/nuccore/NG_007494.1). The identified mutations were compared with the D variants reported in the RhesusBase database (http://www.rhesusbase.info/). There was also a search of the literature to see if there was a link between the genotype found in the samples and the weak and partial D phenotypes that have been described before.

### 2.5 Statistical analysis

The SPSS software, version 23.0 (IBM Corp., Armonk, NY, USA), was used for the analysis of the collected data. The RhD variant frequency was calculated by summing the number of subjects positive for the particular antigen or phenotype and dividing by the total number of subjects. The results were expressed as percentages. The Rh phenotype distribution among RhD samples was presented in tables. Descriptive statistics have also been used. In addition, Chi square values were calculated to compare the frequency of antigens and distribution of haplotypes between different samples. The Fisher’s exact test was used if the chi-square test assumptions were not satisfied.

## 3 Results

### 3.1 Molecular analysis

A total of 479 serological RhD discrepant samples were derived for the study of weak and partial D variants. Molecular analysis confirmed the presence of D antigen variants in 332 samples (69.3%). Of the remaining, 106 (22.1%) were classified as true D negatives and 40 (8.4%) as D positives. In one case, the result was inconclusive. The detected frequency of variants among the discrepant samples was 66% (219/332) weak RhD, 6% (20/332) partial RhD, and 28% (93/332) extremely weak RhD (DEL).

The weak type 2 (n = 91, 27.4%) and RHD*DEL43 variants were the most prevalent (n = 93, 28%). The other most frequently detected specificities were weak type 3 and weak type 48 (n = 28; 8.4%); weak type 1 (n = 27; 8.1%); *DAR1* (n = 19; 5.7%); and *DAR3* (n = 16; 4.8%). **Table 1** summarizes the different variants and the frequencies detected.

**Table 1:** RhD variants frequency among samples with weak or discrepant phenotypes.

RHD allele	n	%
Weak type		
Weak type 1	27	8.1
Weak type 2	91	27.4
Weak type 3	28	8.4
Weak type 17	1	0.3
Weak type 48	28	8.4
Weak type 59	9	2.7
DAR1 (weak partial 4.2)	19	5.7
DAR3 (weak partial D 4.0.1)	16	4.8
Partial D type		
DAU, DAU4, DAU5, DAU5.01	3	0.9
DIIIa, IIIc, III type 4, III type 6	2	0.6
DFR1, DFR3, DFR5 o RHD Ψ	4	1.2
DVI type 1	4	1.2
DVI type 2	1	0.3
DVI type 4	5	1.5
RHD*D-CE(8-9)-D	1	0.3
DEL type		
RHD*DEL43 (W16R)	93	28
**Total**	332	100
RhD negative	106	NA
RhD positive	40	NA
Inconclusive	1	NA

NA. Not applicable because the percentage has been obtained from RhD variants.

### 3.2 Rh phenotyping

Of the total samples entered in this study, 305 were phenotyped for antigens C, c, E, and e. The ccEe (R2r) phenotype was the most frequent (117/305; 38.4%), followed by Ccee (R1r) (81/305; 26.6%), and ccee (rr) (55/305; 18%).

Rh phenotype variants were more common in RHD*DEL43 for the ccEe (R2r) phenotype than the CcEe (R1R2) phenotype (n = 54/62 *vs*. n = 7/62). In the type 1 weak variant, the most frequent Rh phenotype was Ccee (R1r) (n = 18/19; 94.7%), while in the type 2 weak variant it was ccEe (R2r) (54/56; 96.4%). In the weak type 3 variant, the most frequent Rh phenotypes were CCee (R1R1) (n = 9/19; 47.4%) and Ccee (R1r) (8/19; 42.1%). In the weak type 48 variant, the most frequent Rh phenotype was Ccee (R1r) (20/21; 95.2%). **Table 2** summarizes the results of the Rh phenotype in the different variants detected.

**Table 2:** Rh phenotypes distribution among RhD samples.

Variant	Phenotype									
	*CCDee* (R_1_R_1_)	*CcDEe* (R_1_R_2_)	*CcDee* (R_1_r)	*Ccddee* (r’r)	*ccDEE* (R_2_R_2_)	*ccDEe* (R_2_r)	*ccDee* (R_0_r)	*ccddEe* (r’’r)	*ccddee* (rr)	Total
Weak Type										
Weak type 1	0	0	18	0	0	0	1	0	0	19
Weak type 2	0	1	0	0	1	54	0	0	0	56
Weak type 3	9	2	8	0	0	0	0	0	0	19
Weak type 17	0	0	1	0	0	0	0	0	0	1
Weak type 48	0	0	20	0	0	0	1	0	0	21
Weak type 59	0	0	9	0	0	0	0	0	0	9
Weak partial 4.0	0	0	3	0	0	0	8	0	0	11
Weak partial 4.2	0	0	0	0	0	0	8	0	0	8
Partial D type										
Partial D type DAU	0	0	0	0	0	0	1	0	0	1
Partial D III type 6	0	0	0	0	0	0	1	0	0	1
Partial D type DIIIa	0	0	1	0	0	0	0	0	0	1
Partial D DVI type 1	0	0	0	0	0	2	0	0	0	2
Partial D DVI type 4	0	0	3	0	0	0	0	0	0	3
DFR1, DFR3, DFR5	0	0	1	0	0	0	1	0	0	2
DEL type										
RHD*DEL43	0	7	0	0	1	54	0	0	0	62
RhD Negative	0	0	0	3	0	0	0	1	55	59
RhD Positive	1	4	16	0	1	7	0	0	0	29
Inconclusive	0	0	1	0	0	0	0	0	0	1
Total	10	14	81	3	3	117	21	1	55	305

A specific study was carried out because the DEL phenotype in other groups, mostly Asians, is linked to the expression of the C antigen, which is used to find DEL people. In our case, an association is observed between the DEL phenotype and the expression of the c antigen. **Table 3** shows the distribution of RhCcEe antigens among subjects classified as RhD-negative and RHD*DEL43. Most samples phenotyped as RhD-negative and RHD*DEL43 were negative for antigen C and positive for antigens c and e. Antigen E is linked to the RHD*DEL43 variant; it was found in all 62 samples that had this mutation (62/62; p<0.001). By that, it was detected just in one Rh-negative sample.

**Table 3:** Association of RhD phenotypes among RhCEce phenotypes.

RhCEce phenotype	Rh negative	Rh DEL	p value
*ccee*	55	0	<0.001
*ccEe*	1	54	
*Ccee*	3	0	
*ccEE*	0	1	
*CcEe*	0	7	
Total	59	62	

Fisher’s exact test.

## 4 Discussion

In Chile, the typing of the D antigen and its variants is carried out through established protocols for screening donors, patients, and pregnant women. Several centers are still looking into weak and incomplete forms of the D antigen, even when the strategy is used on donors who have anti-D antibodies that recognize the DVI variant and on patients and pregnant women who do not have antibodies that recognize this variant. This is a strategy that allows the characterization and identification of D variants from a serological and molecular point of view (13). The Public Health Institute of Chile has provided guidelines on the studies necessary to recognize these variants in the routine laboratory and their subsequent referral to the National Reference Laboratory. The guidelines say to use at least two anti-D antibodies, one of which should be able to recognize the DVI variants for routine use at room temperature and the other should be an IgG antibody that allows evaluation. Also, it shows how important it is to use molecular biology to study D antigen variants, just like the Association for the Advancement of Blood & Biotherapies and the College of American Pathologists, which recommend RHD genotyping in pregnant women. This step would help make the best use of anti-D immunoglobulin for prevention and the stock of RhD-negative units for patients who are really at risk of getting alloimmunized to the D antigen (14).

The D antigen of the Rh system presents variations, which are classified into 3 large groups: D weak, D partial, and D extremely weak (DEL). Their frequencies vary significantly by race and ethnicity (13). African descendants and individuals of mixed ancestry are more likely to carry RHCE variants than Caucasians and Asians (15–27). Using both in-house and commercial genotyping assays, researchers have investigated the diversity and frequency of RH alleles in blood samples and/or subjects with sickle cell disease at high risk of alloimmunization (15–28). The reported frequencies of RHCE variant alleles are inconsistent among reports, likely due to several reasons, including differences in study design, molecular strategies used for RHCE characterization, and population ethnicity. However, there is agreement regarding the clinical significance of RHCE variants caused by c.48G>C and c.733C>G [RHCE*ce.01 (ce48C), RHCE*ceVS.01 (ce733G), and RHCE*ceVS.02 (ce48C, 733G)].

In this study, we analyzed 479 samples that had different results when they were first tested for the D antigen. These samples were sent to the Reference Laboratory of the Public Health Institute of Chile for serological and molecular confirmation. The most common variants were RHD*DEL43 (n = 93; 28%), weak type 2 (n = 91; 27.4%), weak type 3 (n = 28; 8.4%), weak type 48 (n = 28; 8.4%), and weak type 1 (n = 27; 8.1%). The RHD*DEL43 variant is considered to be very weak (DEL). It is caused by a change from a thymine nucleotide to a cytosine at position 46 of exon 1 of the RHD gene. Also, it is considered a rare mutation and has been reported in the Caucasian populations of Switzerland and Argentina (29). It is associated with the ccEe phenotype, which was also confirmed in our study (**Table 2**).

The frequency of the DEL phenotype varies significantly depending on the geographic location of the study. In the Han population (China), the frequency of DEL is 30% (30, 31). In Japan, only 0.5% of individuals are RhD-negative, and of these, 28% express the DEL phenotype (32, 33). In Korea, 0.15% of the population is RhD-negative, and of these, 17% express the DEL phenotype (34, 35). In Caucasians, the number of people with DEL phenotypes are much lower: only 0.1% of them are RhD-negative and about 15% are RhD-positive (36). In Chile, interestingly, the frequency of RhD negatives is only 5.5% (11), and our study shows a significant presence of the DEL variant among the samples analyzed. However, the samples tested were only those with serologically discrepant results, introducing a selection bias. Therefore, this limitation could be addressed using a larger population in future studies.

**Figure 1:**
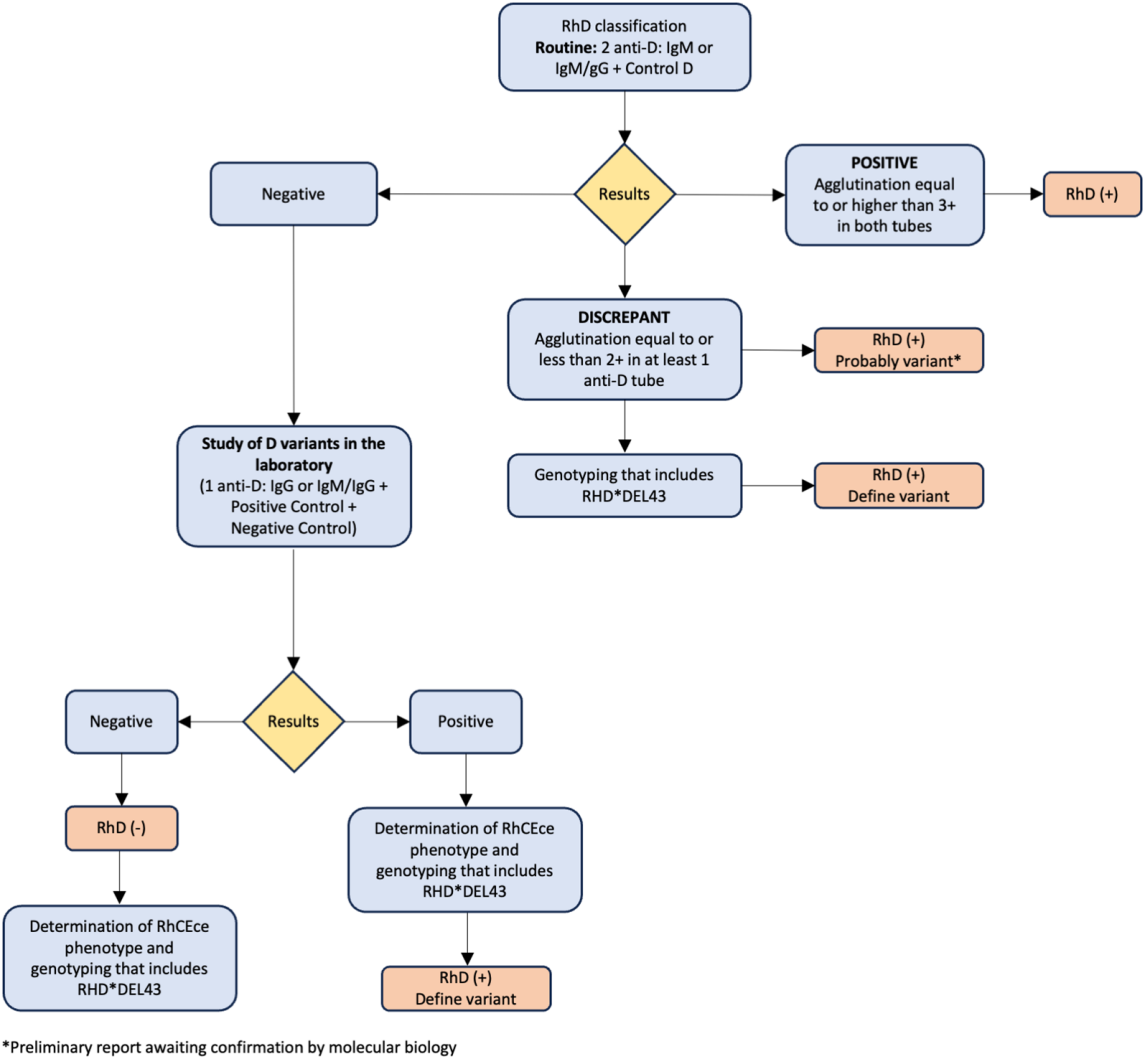
Recommended algorithm for classification of RhD and RhD variants.

By doing molecular typing on people and subjects from many different ethnic backgrounds, scientists have been able to figure out the biological pathways behind Rh symptoms that are changed or not present. To date, more than 200 *RHCE* alleles have been documented in the literature (37–43). The RHCE gene shows genetic diversity through four different molecular mechanisms: (I) single nucleotide changes, (II) insertions, (III) deletions, and (IV) gene rearrangements (gene conversion). These mechanisms can lead to diminished or partial expression of the C, c, E, and e antigens, as well as the emergence of low-prevalence antigens or the absence of high-frequency antigens. Moreover, certain genetic modifications within the *RHCE* gene have the potential to lead to the production of a RhCE protein that lacks functionality. Examples of such mutations are D- -, DC^w^-, Dc-, and D••. Rh_null_ is a condition in which there are no Rh proteins on the membrane of RBCs. This is caused by inheriting RHCE alleles that do not work and RHD that has been deleted (44, 45). Individuals who exhibit varying levels of the RhCE antigen have been found to produce alloantibodies with specificities such as anti-e-like and/or anti-C-like (e.g., anti-hrS, anti-hrB, anti-RH18, and anti-RH34), which are challenging to detect by serological methods. These antibodies, which have been proven to be medically important, are known to trigger transfusion responses. Various combinations of RHCE variations can frequently be passed down along with an RHD variant allele. As a result, these individuals may produce antibodies against RhCE antigens in addition to anti-D (46). **Figure 1** shows our suggested algorithm for the classification of RhD and RhD variants.

The molecular analysis showed that in people with DEL, the RHD gene was still there or there was a partial DEL with the loss of an epitope D (47). DEL variants are caused by changes in the RHD gene structure, the splice site, or exon 9 (48). Currently, 40 alleles have been associated with DEL, with frequencies varying according to ethnic background (49). So, in Eastern Asians, the most common SNP found in people who carry DEL is RHD 1227A (K409K), which causes an abnormal frameshift or exon deletion in transcripts and makes D proteins with weak antigen (30). In Caucasians, on the other hand, the most common DEL alleles include a splice site mutation IVS3 + 1G>A and a missense mutation M295I (50, 51).

When exposed to the Asian-type DEL variant, RhD-negative people can have both primary and secondary immune responses (52, 53). This has led to the development of genotyping strategies in areas where this variant is common. One of them is checking for the C and E phenotypes before the molecular study, because RhDEL is common in C+ and/or E+ and this algorithm is easy to use and doesn’t cost much (54). Because the C antigen lowers the density of the D antigen, this link between the C phenotype (Cc or CC) and DEL makes sense (55). However, the universal application of this strategy should be taken with caution since there are reports of DEL individuals with the cc phenotype. In fact, 8.53% of DEL individuals from the Chinese (Han) population carry the cc phenotype (56). In the same way, the cc phenotype is linked to the RHD*DEL43 variant in our study (p<0.001; **Table 3**).

There is ethnic variation in the E antigen and the presence of the DEL variant. Koreans and Japanese people are more likely to have it (28.6% and 8.4%, respectively). It has been shown that a negative phenotyping result for C and E can reliably predict true D- RBCs (100% positive predictive value; n = 4407) and that RBCs that are negative for anti-D but that are C+ and/or E+ are candidates to be genotyped for DEL (57). In our study, all individuals genotyped as RHD*DEL43 presented the E antigen (**Table 4**). This background reveals the need to develop local studies to understand the behavior of these variants and implement protocols appropriate to each region.

**Table 4:** Distribution of RhCEce antigens in Rh-negative and Rh DEL phenotype.

RhCEce antigens	Rh negative	RHD*DEL43	p value
C Positive	3	7	0.164
C Negative	56	55	
E Positive	1	62	<0.001
E Negative	58	0	
c Positive	59	62	0.236
c Negative	0	0	
e Positive	59	61	1.000
e Negative	0	1	

Fisher’s exact test.

The weak variant types 1, 2, and 3 came after DEL in terms of frequency in the samples that were looked at (27/8.1%; 91/27.4%; 28/8.4%, respectively). These variants are common in Caucasians and rare in Africans and Asians (13). The type 2 weak variant is the second most common in our study population. Similar results were found in the populations of Portugal and Brazil, which can be explained by the fact that there is ethnic variation (58, 59).

An interesting finding of our study is the presence of rare RhD variants. The most frequent of them is weak type 48 (n = 28; 8.4%), mostly accompanied by the Ccee phenotype. A change from guanine to thymine at nucleotide 182 of exon 2 of the RHD gene results in weak type 48. This change is linked to the cE phenotype. Another rarely reported variant, which is associated with the CcEe phenotype and which could be linked to the Chinese population (Han), is D-weak type 59, which was detected in 2.7% of the samples that also expressed the Ccee phenotype. Furthermore, in one case, the weak variant type 17 (n = 1; 0.3%) was identified, which has been described in the Chinese, Korean, and German populations (41).

In relation to partial variants, DVI type 1 (n = 4; 1.2%), DVI type 2 (n = 1; 0.3%), and DVI type 4 (n = 5; 1.5%) were detected. DVI type 4 presents a frequency of 50% among the DVI variants in our study. This agrees with what has been reported in the Spanish population, in which DVI type 4 corresponds to 65% of the total DVI variants. This result is expected, given our shared genetic ancestry with the Spanish population (60).

One interesting thing about this study is that the variants that were found the most, weak type 2 and RHD*DEL43, are linked to the ccDEe (R2r) phenotype. The frequencies found in our population might be able to explain this. On the one hand, the type 2 weak variant would be due to the Caucasoid component (61), while the RHD*DEL43 variant would be related to the aboriginal component, as occurred with its discovery in Argentina (29). In our study, the percentage of RHD*DEL43 variant cases (93; 28%) was higher than those described in Switzerland and Argentina (29, 61). Apparently, this DEL variant would be related to the ethnic origin of our population; however, future studies are required to verify this hypothesis.

## 5 Conclusions

The indirect antiglobulin test used in Chile can find weak variants but not the DEL variant. This means that donors might be mistakenly thought to be Rh-negative. This way, D antigen phenotyping should be able to find all D antigen carrier donors who might be able to help people get immune to this antigen and keep D-negative or D-variant patients from getting immune to it. It should also prevent the needless use of D-negative units and antenatal Rh immunoglobulin.

We analyzed a group of discrepant samples for RhD antigen detection derived from the Immunohematology Reference Laboratory of the Public Health Institute of Chile. The most common variants detected in this group were DEL type (RHD*DEL43) and weak (weak type 2), which are linked to the ccDEe (R2r) phenotype. This study provides preliminary information that suggests the need to perform genotyping of those donors who are typed as RhD-negative in conventional immunohematological screening. Therefore, in the future, it should be included in conventional screening.

## Data availability statement

The datasets presented in this study can be found in online repositories. The names of the repository(s) and accession number(s) can be found below: GenBank accession numbers are OR862777-OR862869, OR862870-OR862900, OR862901, OR862902-OR862904 and OR862905-OR862906.

## Ethics statement

Ethical approval was not required for the studies involving humans because This study retrospectively reviews the medical records of patients who have undergone a specific procedure for clinical purposes. The studies were conducted in accordance with the local legislation and institutional requirements. The human samples used in this study were acquired from gifted from another research group. Written informed consent to participate in this study was not required from the participants or the participants’ legal guardians/next of kin in accordance with the national legislation and the institutional requirements.

## Author contributions

AA: Conceptualization, Investigation, Supervision, Writing – original draft, Writing – review & editing. DZ: Conceptualization, Investigation, Supervision, Writing – original draft, Writing – review & editing. ER: Investigation, Supervision, Writing – review & editing. JF: Investigation, Supervision, Writing – review & editing. GB: Investigation, Supervision, Writing – review & editing. FP: Investigation, Supervision, Writing – review & editing. SC: Writing – original draft, Writing – review & editing. NS: Writing – original draft, Writing – review & editing. CS: Writing – original draft, Writing – review & editing. JO: Investigation, Supervision, Writing – review & editing. JC: Conceptualization, Writing – original draft, Writing – review & editing.
